# Conditional and Unconditional Cash Transfers to Improve Use of Contraception in Low and Middle Income Countries: A Systematic Review

**DOI:** 10.1111/sifp.12004

**Published:** 2016-11-17

**Authors:** M. E. Khan, Avishek Hazra, Aastha Kant, Moazzam Ali

## Abstract

This systematic review synthesizes evidence on the impact of conditional and unconditional cash transfers (CCT and UCT) on contraception in low‐ and middle‐income countries. Scientific and gray literature databases were searched from 1994 to 2016 and 11 papers from ten studies were included. Most of the studies had low risk of bias. Cash transfers were used for increasing school attendance or improving health and nutrition, but not directly for contraception. Three studies showed positive impact on contraceptive use and four showed a decrease in fertility outcomes. An increase in childbearing was observed in two studies, and three studies demonstrated no impact on fertility indicators. All studies treated contraceptive use or fertility only as unintended and indirect outcomes. The available evidence on impact of CCT and UCT on contraception is inconclusive due to the limited number of studies, varying outcome measures, and lack of intervention specifically for contraception.

Financing health care is an increasing concern, particularly to ensure that people in low‐ and middle‐income countries (LMICs) are able to choose, obtain, and use high‐quality contraceptives. FP2020 and other initiatives have stimulated efforts to revive investment in family planning. Many bilateral, multilateral, and private foundation donors have identified knowledge gaps that could be filled by collective efforts to meet the current unmet need for contraception. One such gap was lack of evidence on the role of financial mechanisms in influencing contraceptive use (Ali et al. 2014; Askew and Brady 2013). A systematic review of literature on financing mechanisms, such as conditional and unconditional cash transfer programs, could create an evidence base measuring the impact of such programs on use of contraception.

The primary objective of conditional cash transfer (CCT) programs is to alleviate poverty by giving money to poor people in return for fulfilling specific behavioral conditions (Doetinchem et al. 2008) such as gaining access to maternal and child health care services and educational opportunities, while unconditional cash transfer (UCT) programs do not require any conditions on the part of recipients (Arnold et al. 2011). Such financial assistance to poor households may improve health outcomes by making health care, food, or education more affordable by enhancing household income in the short term and human capabilities in the medium and long term (Bourguignon et al. 2004; Arnold et al. 2011). Since 1997, countries in Latin America and the Caribbean have implemented and evaluated CCT programs with health components. Subsequently, many developing countries in other regions also introduced cash transfer programs.

Research on cash transfers has looked at various behavioral, health, and educational outcomes. Cash transfer programs were found to be effective in increasing the use of preventive services, improving antenatal care services and immunization coverage, improving health and nutritional status, and reducing HIV risks (Gertler 2004; Rawlings and Rubio 2005; Lagarde, Haines, and Palmer 2007, 2009; Arnold et al. 2011; Ranganathan and Lagarde 2012; Pettifor et al. 2012; Glassman et al. 2013; Morgan et al. 2013; Carvalho et al. 2014). There is, however, a paucity of systematic reviews critically assessing the impact of cash transfer programs on family planning.

This systematic review synthesizes evidence on cash transfers for family planning and their impact on contraceptive use and related outcomes in LMICs. The specific objectives are to review and identify areas where the impact of CCT and UCT on contraception is strong; and identify the current gaps in knowledge and potential research topics in health care financing using CCT or UCT to promote the adoption of contraceptive methods.

## METHOD

### Search Strategy

This systematic review was conducted in accordance with WHO guidelines. The following eligibility criteria were used for preliminarily screening of literature: (1) the title and abstract or executive summary of the paper was available in English, (2) the study was conducted in or after 1994, (3) the study was conducted in low‐ and middle‐income countries, and (4) the study assessed the impact of a CCT and/or UCT intervention on contraceptive use and/or fertility.

A comprehensive search strategy was developed and tested as part of the formal database screening. Search strategy terms were identified, search term blocks were constructed, and the main search strategy was implemented using a combination of search term blocks and filters. A systematic review of peer‐reviewed literature was undertaken by searching the following medical and social science databases: PubMed/MEDLINE; Cochrane Library; Popline; IDEAS: Economic and Finance Research; Index Medicus for South‐East Asia Region (IMSEAR); WHO library database (WHOLIS); Index Medicus for the WHO Eastern Mediterranean Region (IMEMR); Western Pacific Region Index Medicus (WPRIM); African Index Medicus (AIM); American Association of Critical‐Care Nurses (AACN); and Literatura Latino Americana em Ciências da Saúde (LILACS).

Two search strategies were adopted. The first employed general keywords like “conditional cash transfers” and “unconditional cash transfers” along with key terms for contraceptive use, family planning, and developing countries. The second strategy streamlined the process through another independent search with keywords provided for contraceptive use. Subsequent searches were done in combination with search terms for contraceptive use along with “conditional cash transfers” and “unconditional cash transfers.” Additional key terms like “cash incentives,” “cash benefits,” “demand side financing,” “supply side financing,” and “financing mechanism” were also added to the search. The search for papers to be included in this review was concluded in May 2016.

Following the formal database screening, several online resources and institutional sites were searched and relevant reports were identified. Simultaneously, cross‐referencing was carried out to identify other relevant studies. The websites of the following organizations, institutions, and associations were searched for gray literature: Population Council, World Bank, Web of Sciences, International Conference on Family Planning, International Union for the Scientific Study of Population (IUSSP), Population Association of America (PAA), Department for International Development (DFID), United States Agency for International Development (USAID), Harvard University, University of California‐Berkeley, greylit.org, George Washington University, Guttmacher Institute, London School of Hygiene and Tropical Medicine (LSHTM), and Canadian International Development Agency (CIDA).

### Inclusion Criteria

We included quantitative studies with either a control or credible counterfactual as part of the following study designs: randomized control trials (RCTs), controlled clinical trials, controlled before and after (CBA) studies, interrupted time series studies, and cohort or longitudinal studies.

The population for the systematic review included women, adolescents, married and unmarried, in all age groups, living in LMICs. The interventions included conditional and unconditional cash transfers to beneficiaries, their parents or guardians, families, or female‐headed households, upon fulfilling certain health‐ and education‐related conditions for CCTs, or without any conditions for UCTs.

The aim of the systematic review was to measure the effect of conditional cash transfers and unconditional cash transfers on one or more of the following primary outcomes: use of contraceptive services or commodities, method continuation and/or switching, new contraceptive users, contraceptive prevalence rate, unmet need for modern contraceptive methods, and method mix. We also included papers that addressed one of the secondary outcomes, namely fertility (e.g. timing of first birth, birth spacing/interval, ever pregnant). The literature search and screening of articles were focused on finding studies that included either contraceptive use or fertility. In other words, studies that examined fertility outcomes but not contraceptive use were also included. The other secondary outcomes were health outcomes (e.g. STIs, maternal mortality reduction); incidence of contraceptive side effects; changes to organizational services/efficiency (e.g. stock outs); equity; financial risk protection; cost, cost‐efficiency, and cost‐effectiveness; sustainability; acceptability/satisfaction of services; quality of care and services; and scaling up.

### Exclusion Criteria

Studies that did not have either a control or credible counterfactual, or did not include either primary outcomes or fertility outcomes, were excluded from the systematic review.

### Selection of Included Studies

Two independent reviewers conducted title and abstract screening, full paper screening, data abstraction, and methodological quality rating. Disagreements were resolved by consensus between the two reviewers or, if consensus could not be reached, by a third independent reviewer.

### Data Extraction

A standardized data extraction form, provided by WHO, was used to extract required information from the included studies. A database was created in Microsoft Excel, separately for literature retrieved through formal and gray literature database searches.

### Data Analysis

The studies were summarized in tables to assess the effectiveness of the interventions, based on the difference‐in‐differences between the intervention and control areas. Given the diversity of indicators across studies, a broad range of outcome indicators was included. If a study presented measures of statistical significance, this was taken into account in determining effectiveness. Because the number of selected studies was limited and the outcome measures were diverse, neither meta‐analysis nor sensitivity analysis was conducted.

### Quality Assessment and Risk of Bias in Studies

Quality rating for each of the studies was performed according to the standard criteria recommended by Effective Practice and Organization of Care (EPOC) (Cochrane Effective Practice and Organization of Care 2009a) and the *Cochrane Handbook for Systematic Reviews of Interventions* (Higgins and Green 2011). The studies that used RCT or CBA research designs were assessed and rated to have low, unclear, or high risk of bias according to: Adequately generated allocation sequence, Adequately concealed allocation, Similar baseline outcome measurements, Similar baseline characteristics, Adequate addressing of incomplete outcome data, Adequate prevention of knowledge of allocated interventions, Adequate protection against contamination, Freedom from selective outcome reporting, and Freedom from other risk of bias. The percentage of the reviewed studies with specific category of risk of bias (low, unclear, and high) for each of the above‐mentioned parameters was calculated.

Following the EPOC criteria (Cochrane Effective Practice and Organization of Care 2009b), assessment of the cohort studies was based on answers to eight questions: Was selection of exposed and non‐exposed cohorts drawn from the same population? Can we be confident in the assessment of exposure? Can we be confident that the outcome of interest was not present at the start of the study? Did the study match exposed and unexposed populations for all variables associated with the outcome of interest, or did the statistical analysis adjust for these confounding variables? Can we be confident in the assessment of the presence or absence of confounding factors? Can we be confident in the assessment of outcomes? Was the follow up of cohorts adequate? and Were co‐interventions similar between groups? We based our risk assessment on one of four possible answers to each of the aforementioned questions: definitely yes (low risk of bias), probably yes, probably no, and definitely no (high risk of bias).

Each of the reviewers independently assessed the risk of bias for each study. The divergences of grading were resolved by discussion. After quality assessment, studies that were rated low in the majority of the risk of bias parameters were assessed to have strongest quality of evidence.

### Protocol Registration

The protocol was registered with PROSPERO (CRD42016039791), available at http://www.crd.york.ac.uk/prospero/display_record.asp?ID=CRD42016039791

## RESULTS

### Study Selection

Using 27 search engines, a total of 231 citations were found to be relevant to the subject under review. After removing 141 duplicate citations, 90 abstracts were read. For papers with multiple publications, or available in both a peer‐reviewed journal and a working paper, the more comprehensive version was used for the review. Further, 28 papers were rejected after reading the abstract, where neither contraceptive use nor fertility outcomes were mentioned. Thereafter, 51 papers were excluded after reading the full text, as they did not satisfy the inclusion criteria. Finally, 11 papers that described data and results from ten studies were selected for the final review (see Appendix Table 1).^1^

### Characteristics of the Selected Studies

The ten studies in this systematic review were published between 2007 and 2016; six were published in 2011 or later. The studies were from seven countries—Honduras, Kenya, Malawi, Mexico, Nicaragua, South Africa, and Zambia—with seven different CCT or UCT programs. All of these cash transfers programs focused on improving health and education outcomes in low‐ and middle‐income countries. The key dependent variables in the studies included current use of modern contraceptive methods (3 studies), current use of any contraceptive method (3 studies), birth spacing (3 studies), teenage pregnancy (1 study), birth in the year preceding the survey or currently pregnant (1 study), ever pregnant (3 studies), and number of children ever born (2 studies). Studies could have more than one dependent variable.

### Risk of Bias

We categorized the risk of bias in the eight studies that used RCT or CBA research designs. The assessment of the risk of bias for adequate generation of allocation sequence revealed that two studies were rated at high risk of bias, while the other six were at low risk. The second parameter, allocation concealment, was unclear in five studies, while two were at high risk and one was at low risk. The baseline outcomes between study groups were similar in five studies, the baseline characteristics were similar in six studies, and incomplete data were adequately addressed in four studies, and in each case these studies were categorized as low risk. Knowledge of allocated interventions was adequately prevented in one study (low risk) and was unclear in the other seven. On the aspect of contamination, two studies had low risk of bias, five were unclear, and one study had high risk of bias. All eight studies were found to have a low risk of selective outcome reporting bias. For the final parameter, other risk of bias, seven studies were rated low, while one was at high risk. In summary, the majority of the reviewed studies had a low risk of bias.

The study‐specific risk of bias, indicating the quality of evidence, is given in Appendix Table 1. Our risk assessment for the two cohort studies revealed that both had a low risk of bias for four or more domains of the risk of bias tool. Of the ten reviewed studies, two (Stecklov et al 2007; Feldman et al. 2009) that were rated low in seven of the nine parameters of risk of bias were assessed to have strongest quality of evidence.

### The Interventions and Their Impact

Of the ten selected studies that explored the impacts of CCT and UCT programs on contraceptive use or fertility, four were from Mexico, with the *Oportunidades* program as the CCT intervention (Lamadrid‐Figueroa et al. 2008; Feldman et al. 2009; Darney et al. 2013; Arena et al. 2015); one from Nicaragua that focused on *Red de Protección Social* (RPS) as the CCT intervention (Todd et al. 2012); one (Stecklov et al. 2007) was a comparative study of three CCTs—PROGRESA (Mexico), RPS (Nicaragua), and *Programa de Asignación Familiar* (PRAF) (Honduras); one from Malawi, with the *Zomba* cash transfer program as the CCT (Baird et al. 2010; Baird, Craig, and Ozler 2011); one from Zambia, with Child Grant Programme (CGP) as a UCT intervention (Palermo et al. 2016); one from Kenya, with unconditional cash transfers for orphans and vulnerable children (CT‐OVC) (Handa et al. 2015); and one from South Africa, with Child Support Grant (CSG) as the UCT program (Rosenberg et al. 2015).

The CCT program *Oportunidades* in Mexico began in 1997 as PROGRESA and was renamed in 2002. This program transferred cash to female‐headed households, or to wives of household heads, contingent upon children's minimum 85 percent attendance at school, pregnant women's attendance at five antenatal visits and two postnatal visits, all family members’ attendance at a health center checkup, and female household head's attendance at health talks. The cash transfers varied according to age and children's gender, with larger payments for girls in higher education grades. The monthly grants ranged from about US$11 in the third grade of primary school to about $58 for boys and $66 for girls in the third year of high school. The nutrition component included a fixed monthly transfer of about $16 for improved food consumption as well as nutritional supplements (Shanghai Poverty Conference nd; Stecklov et al. 2007; Lamadrid‐Figueroa et al. 2008; Darney et al. 2013; Feldman et al. 2009; Arena et al. 2015).

Of the studies that evaluated the *Oportunidades* program, one revealed a 5 percentage point increase over three years in contraceptive use among young women aged 20–24 years, with no impact on adolescents aged 15–19 (Lamadrid‐Figueroa et al. 2008). The study also revealed an increase of 20 percentage points in the prevalence of contraceptive use due to the CCT intervention among women from the poorest households. The amount transferred was a major incentive for influencing household members’ behavior; *Oportunidades* influenced the extremely poor the most, as that population segment showed the greatest commitment to the program for increasing their real incomes. Darney et al. (2013), however, revealed that exposure to *Oportunidades* was not independently associated with current use of modern contraceptive methods among adolescents or young women. The program had no direct effect on adolescent pregnancy. Feldman et al. (2009) found that modern contraceptive use increased by 18 percentage points between 1998 and 2003 among women in the intervention area and by 10 percentage points in the control area. The cohort study by Arena et al. (2015) noted a significant negative effect of the program: fertility, measured in terms of number of children ever born, increased by 5 percent from pre‐program levels.

Nicaragua's RPS program began in 2000 and offered two types of CCTs, on nutrition/health and education, contingent on compliance with a set of conditions. The conditions for nutrition/health were mother's attendance at bi‐monthly health workshops or talks, attendance at regular age‐specific health checkups, and up‐to‐date vaccinations for children under five years of age. The conditions for education were enrollment and regular attendance of all children aged 7–13 years who had not completed fourth grade. The cash transfers were delivered bi‐monthly to mothers (or principal household females). For the health component, $224 per year per household was paid on meeting the eligibility criteria. The amount of cash transfers for education was approximately $112 per household per year (Stecklov et al. 2007; Todd et al. 2012; Barham et al. 2013).

The study evaluating the RPS program showed that the coefficients for the interaction of treatment and time indicated a lower probability of giving birth and lower total parity among women in the program compared to women in the control group, although neither difference was statistically significant (Todd et al. 2012). The authors also found that the program reduced birth hazards in each of its four time periods by approximately 32 percent.

PRAF in Honduras, which began in 1999, provided CCT to members of poor households who adhered to the following conditions: regular health checkups for pregnant women and children under three years of age, and school enrollment and regular attendance for children aged 6 to 12 years who had not completed fourth grade. Further, the intervention was valid only for households with three or fewer children. To implement the CCT program, funds were transferred to health and education facilities for child growth and development monitoring and for vaccinations at public health facilities. For nutrition and health care, a transfer of $48 per person per year was made to eligible households during a woman's pregnancy and for a post‐partum check‐up. For the education component, children were given approximately $38 per year (Stecklov et al. 2007; Galiani and McEwan 2013).

The comparative study of PROGRESA (Mexico), PRAF (Honduras), and RPS (Nicaragua) showed a negative impact of PRAF. Childbearing increased in the intervention area in comparison to the control, with the probability of a woman giving birth in a given year increasing by an average of 1.7 percent, and the probability of a birth or current pregnancy increasing by 3.9 percent. There was no impact of either RPS or PROGRESA on fertility (Stecklov et al. 2007).

Malawi's *Zomba* Cash Transfer Program, started in 2007, was a combination of a CCT and UCT program. Within the CCT component, the cash transfer included monthly incentives in the form of school fees and cash transfers to beneficiaries (school‐going girls and recent dropouts) and their parents or guardians for regular school attendance; for girls attending secondary school, the fees were paid directly to the school. For UCT, there was no requirement to attend school to receive the monthly cash. Attendance was not checked for recipients of this arm and they received payments by being present at the transfer locations every month. The amount of transfer varied between $1 and $5 per month for the beneficiaries and between $4 and $10 per month for their parents or guardians. The amount was determined through a random process across the enumerated area to ensure that each parent received the same offer (Baird et al. 2010; Baird, Craig, and Ozler 2011).

The two papers that evaluated the impact of Malawi's *Zomba* program, in which schoolgirls enrolled at baseline were followed up for one year (Baird et al. 2010) and for two years (Baird, Craig, and Ozler 2011), revealed that the program had little or no impact on the incidence of childbearing in the CCT arm while a large decline in pregnancy was observed in the UCT arm. The likelihood of pregnancy was reduced by 6.7 percentage points in the UCT arm.

The Zambian CGP, a UCT program to reduce extreme poverty, was started by Zambia's Ministry of Community Development, Mother and Child Health in 2010. The program had specific objectives of increasing school attendance, reducing under‐5 mortality and morbidity, increasing household asset ownership, and increasing the number of households having a second meal per day. A sum of $12 was distributed bi‐monthly to the female caregiver for children aged 0–5 years (Palermo et al. 2016).

The evaluation by Palermo et al. (2016) showed that after 48 months of the intervention, contraceptive use had increased within both study groups (by 37–54 percent among treatment group women and 39–51 percent among control group women), which corresponded to the overall increase in contraceptive use at the national level. No program impact on modern contraceptive use was observed at 36 or 48 months. At 24 months, the treatment group women were 2.5 percentage points less likely to have ever been pregnant. At 36 months, the program had a positive impact in reducing fertility among women aged under 25 years: young women in treatment households had 10 percent fewer births than those in control households.

Another UCT program, the CT‐OVC, is an antipoverty program implemented by Kenya's Ministry of Gender, Children and Social Development in 2007. Eligible households—those who were ultra‐poor (in the lowest expenditure quintile: $15) and had at least one orphan or vulnerable child under age 18 years, with at least one deceased parent or a parent or main caregiver who was chronically ill—receive US$20 per month. The program is unconditional, although households are informed that the care and protection of the resident OVC is their responsibility (International Policy Centre for Inclusive Growth 2012; Handa et al. 2015). The study by Handa et al. (2015) on the UCT's impact on pregnancy revealed that it reduced the likelihood of pregnancy over a four‐year period among women aged 12–24 years who had never given birth at baseline by 5 percentage points, or 34 percent.

The CSG, South Africa's largest social protection program, began in 1998 and provides UCT to children living in households below a poverty threshold in which there is a child aged less than seven years. The CSG expanded the eligibility age for children up to age nine in 2003, age 11 in 2004, age 14 in 2005, age 15 in 2009, and age 18 in 2010. The grant payment was US$8 initially and increased to $27 per month per child (Rosenberg et al. 2015). A cohort study that evaluated the CSG program showed that receipt of the UCT was significantly associated with lower second pregnancy rates (Rosenberg et al. 2015). Time to second pregnancy was significantly longer among CSG recipients compared to non‐recipients.

## DISCUSSION

The studies reviewed here showed that cash transfer programs were used to improve maternal and child health and child education, thereby increasing children's quality of life. Stecklov et al. (2007) argued that CCT programs, by their effects on both income and prices of commodities and services, have the potential to affect the relationship between child quality and quantity and, thus, fertility. Two studies documented a negative impact of cash transfer programs on fertility outcomes and showed an increase in childbearing (Stecklov et al. 2007; Arena et al. 2015). Four studies showed a positive impact on fertility outcomes measured through increased birth spacing and a reduction in the likelihood of becoming pregnant (Todd et al. 2012; Handa et al. 2015; Rosenberg et al. 2015; Palermo et al. 2016). Three studies demonstrated no impact of cash transfers on fertility indicators (Stecklov et al. 2007; Darney et al. 2013; Baird et al. 2010; Baird, Craig, and Ozler 2011). Three showed a positive impact on contraceptive use (Lamadrid‐Figueroa et al. 2008; Feldman et al. 2009; Palermo et al. 2016).

The studies indicate that CCT and UCT interventions predominantly focused on health, education, and nutrition aspects of women or households. The conditions used were diverse, such as adherence to prenatal and postnatal visits for pregnant women, children's timely vaccinations and growth monitoring visits, mother's attendance at bimonthly health workshops, children's enrollment and regular attendance at school. The programs targeted children and women of reproductive age from low‐income families, aiming to improve direct outcomes such as school enrollment and maternal health. None of the reviewed studies revealed that CCT or UCT was specifically intended to increase contraceptive use or reduce fertility. All selected studies assessed the impact of CCT and UCT on contraceptive and fertility outcomes that are indirectly related to health, nutrition, and education. Stecklov et al. (2007) referred to such outcomes as “unintended outcomes” of the cash transfer programs.

Of the 51 papers that did not meet the review inclusion criteria, only one (Pratinidhi and Lale 2014) used CCT for birth spacing. The program, Second Honeymoon Package (SHP), was a community‐based intervention begun in 2007 in Satara district of Maharashtra, India. Under the SHP, cash transfers were made to couples conditional on delaying their first child by two or three years following marriage. Couples who postponed having children for two years were offered a cash incentive of approximately $75 and those who postponed for three years were offered approximately $113. The study, which evaluated CCT's effect on use of contraception among SHP participants, showed that 92 percent of participants were using modern methods of contraception compared with 18 percent in the control group, with condoms (88 percent) as the most popular method, followed by oral pills (10 percent).

CCT or UCT programs could address the financial barriers for uptake of family planning services and improve access. If such interventions include the supply side as an integral part of implementation, they could improve clinical care at health facilities and increase availability of contraceptive methods or services. These steps could reduce the out‐of‐pocket spending and increase access to family planning services. Ultimately, couples’ demand for family planning services could be met, unintended pregnancies could be avoided, and couples could meet their desired family size.

Broadening the focus of CCT or UCT programs could translate the current indirect outcomes into direct outcomes. With a strict conditionality of cash transfers for having no more than two children, the negative motivation to increase fertility could be eliminated, leading to direct desired outcomes of lower birth rates, increased child spacing, increased contraceptive use, and reduced fertility. The SHP study showed that a CCT for birth spacing could provide a positive result. Stecklov et al. (2007) identified three factors most likely to affect CCTs for fertility—amounts transferred to households, how programs influence absolute and relative costs associated with having and investing in children, and how a CCT program influences supply‐side factors like contraceptive availability at public facilities.

Our systematic review shows that only a few studies have assessed impacts of conditional or unconditional cash transfers on increasing contraceptive use or decreasing fertility. The studies that documented a positive impact considered contraceptive use only as an indirect and unintended outcome. There is, therefore, a major knowledge gap in our understanding of the impacts of conditional and unconditional cash transfers on contraception and family planning. The systematic review flags a need to address the relationship between CCTs or UCTs and contraceptive use and contraceptive prevalence rates. The review also identified several research gaps, such as whether and how the cash transfer programs influence contraceptive use patterns, delaying the first child, and method switching.

### Limitations

Only ten studies were included in this systematic review. We conducted an exhaustive search of the literature to identify any relevant study that satisfied the inclusion criteria, but it is possible that some studies were missed. We faced methodological challenges during the systematic review. The search strategy was too robust and not suitable for many databases. Many search engines and websites did not accept more than seven words; in such cases, the key words were modified, and only broad terms were used. The stringent screening questions led to rejection of many good descriptive papers. Synthesizing the results of the studies was difficult because studies used different indicators for measuring contraception and fertility.

## CONCLUSION

There is a dearth of well‐designed studies focusing primarily on the impact of CCT or UCT programs on contraceptive use, increased child spacing, and the feasibility of sustaining and scaling up CCT or UCT interventions. Thus, the available evidence on the impact of cash transfers on contraception is inconclusive. FP2020 and the Sustainable Development Goals provide a major opportunities for intervention studies using a mixed‐method approach that could examine the direct impact of CCT or UCT on contraceptive use with scientific rigor. It is also important to document the pathways through which changes in contraceptive use or fertility occur. Findings of such studies and the strategies could be promoted to help achieve a sustained development agenda.

## Supporting information

Disclaimer: Supplementary materials have been peer‐reviewed but not copyedited.

**Appendix Table 1**. Details of the 11 papers from the ten reviewed studies

## Footnotes

1 Appendix table is available at the supporting information tab at wileyonlinelibrary.com/journal/sfp.
