# Supplementary material for: Conditional and Unconditional Cash Transfers to Improve Use of Contraception in Low and Middle Income Countries: A Systematic Review
Source: Stud Fam Plann. 2016 Nov 17;47(4):371–83. doi: 10.1111/sifp.12004 (PMC5434941; doi:10.1111/sifp.12004)
Supplement: Supplementary file 1 — Appendix Table 1. Details of the 11 papers from the ten reviewed studies [file SIFP-47-371-s001.docx]

**Appendix Table 1: Details of the 11 papers from the ten reviewed studies**

|  | **Study (Author, Year, Location)** | **Intervention** | **Study Design, Study Period and Study Size** | **Outcome measures** | **Main Findings** | **Risk of bias** |
| --- | --- | --- | --- | --- | --- | --- |
| 1.1 | Arena et al. 2015  Mexico | *Oportunidades* Program | Longitudinal study with treatment and comparison group  Data from Mexican Family Life Survey (MxFLS). First wave in 2002, second wave during 2005-2006 and third wave in 2009.  The study sample consists of women in rural areas between the ages of 10 and 40 before the program started (1997) and were interviewed in all three years of the MxFLS.  Final sample size 1,650 women. | Number of children born and pregnancies | - The size of the significant and positive effect on number of children born and on pregnancies was 0.2, 5 percent increase from pre-program. . - The impacts were of similar magnitude and significance levels during 2005 and 2009. | Total 8 parameters;   - Low : 4 - Unclear (Probably yes or probably no): 2 - High: 2 |
| 1.2 | Darney  et al. 2013  Mexico | *Oportunidades* Program | CBA  1992 survey (N=277,552) that provided data prior to *Oportunidades* implementation; 2006 survey (N=142,961) that contained data about exposure to *Oportunidades* and 2009 survey (N=343,887), which was the most recent DHS.  Analysis was based on 11,138 women aged 15-24 in 1992; 3,832 women aged in 2006 and 6,363 women in 2009 | Lifetime experience  of a pregnancy  and current use  of a contraceptive method | - Current use of any contraceptive method increased by 3 percentage points from 1992-2006 and 6 percentage points till 2009. - Exposure to *Oportunidades* not independently associated with current use of modern contraceptives among adolescents or young adult women - No effect on adolescent pregnancy | Total 9 parameters;   - Low: 4 - Unclear: 2 - High: 3 |

| 1.3 | Feldman  et al. 2009  Mexico | *Oportunidades* Program | RCT  The government scheduled a group of communities to receive the benefits immediately and the rest at a later date. This enabled the program to select treatment and control groups randomly.  Data for the contraceptive-use analysis were obtained from three surveys: the 1998 baseline ENCEL, and the 2000 and 2003 ENCEL fertility modules.  8,568 women in 1998, 6,157 women in 2000 and 1,737 in 2003 | Current use of modern contraceptive method, current use of any contraceptive method, and birth spacing | - During 1998-2003, modern contraceptive use increased from 37 percent to 55 percent among women from intervention area, and from 39 percent to 49 percent in the control area. - From 1998 to 2000, modern contraceptive use increased significantly among women from intervention area than in control area (log odds=0.16, p=0.02). No differential increase between baseline and 2003 or between 2000 and 2003. | Total 9 parameters;   - Low: 7 - Unclear: 2 - High: 0 |
| --- | --- | --- | --- | --- | --- | --- |
| 1.4 | Lamadrid-Figueroa  et al. 2008  Mexico | *Oportunidades* Program | RCT  In 1997, 4004 women aged 12-21 were interviewed. 1480 responded to contraceptive use question.  In 2000, 2346 women aged 15-19 and 2230 aged 20-24 were interviewed.  812 women were common in both 1997 and 2000 | Woman’s current use  of modern contraceptive methods | - No program impact on contraceptive method use among adolescents aged 15-19 years - A 5 percentage point increase in the contraceptive method use among young adult women aged 20-24 years (p<0.05) - Panel data revealed that for women aged 25-29 years, an 8 percentage point increase in contraceptive use among treatment communities than in control. The program impacted the poorest the most, contraceptive use increased by more than 20 percentage points. | Total 9 parameters;   - Low: 6 - Unclear: 2 - High: 1 |
| 2.1 | Todd et al. 2012  Nicaragua | *Red de Proteccion Social* (RPS) program | CBA  2000, 2001, 2002 and 2004  1,581 households were surveyed in 2000 and 1,259 were interviewed in all four surveys.  Analysis based on 881 women (ages 14-45) in 2000 head or spouse of household head at baseline and were observed in 2004 | Birth spacing | - A relatively lower probability of having a birth and to lower total parity relative to the control group, although neither is significant. | Total 9 parameters;   - Low: 3 - Unclear: 3 - High: 3 |

| 3.1 | Baird et al. 2011  Malawi | *Zomba* Cash Transfer Program | RCT  Round 1: October 2007 - January 2008  Round 2: October 2008 - February 2009  Round 3: February - October 2009  Of 176 enumerated areas (EAs), 88 formed treatment group (baseline schoolgirls) and 88 formed control group (baseline dropouts).  Within treatment group, 1,495 girls, divided into CCT arm (506 girls in 46 EAs) and UCT arm (283 girls in 27 EAs) | Pregnancy | - The likelihood of ever been pregnant was reduced by 6.7 percentage points (or 27%) in the UCT arm as compared to control group and CCT arm | Total 9 parameters;   - Low: 6 - Unclear: 3 - High: 0 |
| --- | --- | --- | --- | --- | --- | --- |
| 3.2 | Baird et al. 2010  Malawi | *Zomba* Cash Transfer Program | RCT  Baseline: October 2007 - January 2008  Endline: October 2008 - February 2009  Intervention: December 2007 - January 2008  Panel data: total sample: 2,692 girls  Baseline dropouts: 396 girls in treatment,  408 girls in control  Baseline schoolgirls: 480 girls in treatment,  1,408 girls in control | Teen pregnancy | - The girls of treatment group were 5% point less likely to become pregnant over 1 year (p<0.05) - No impact on the incidence of childbearing at follow up for baseline schoolgirls | - Same as above |
| 4.1 | Stecklov  et al. 2007  Mexico  Hondurous  Nicaragua | PROGRESA  PRAF  RPS | RCT  PROGRESA: 8,817 women   - Baseline: 1997   Endline: 1999  PRAF: 6,456 women   - Baseline: 2000   Endline: 2002  RPS: 2,409 women   - Baseline: 2000   Endline: 2002 | Birth in the 12 months preceding the follow up survey  and whether woman was currently pregnant | - PRAF (Honduras): probability of birth in a given year increased by an average of 1.7 percent points (p=0.091) and probability of a birth or current pregnancy increased by 3.9% (p=0.002) for women in treatment community than in control - No impact of either RPS (Nicaragua) or PROGRESA (Mexico) on fertility | Total 9 parameters;   - Low: 7 - Unclear: 2 - High: 0 |
| 5.1 | Palermo et al. 2016  Zambia | Zambian Child Grant Programme (CGP) | RCT  2,515 beneficiaries and non-beneficiaries households belonging to 90 community welfare assistance committee, divided as 45 as treatment and 45 as control.  Baseline survey: October to November 2010  Follow up surveys:  At 24 months: October to November 2012  At 36 months: October to November 2013  At 48 months: October to November 2014  At 36 month and 48 month follow up surveys, information on current use of contraceptives was collected. | Use of any contraceptive method, use of modern contraceptive methods and number of children ever born to a woman | - By 48 months, a net increase of 5 percentage points in contraceptive use among treatment women as compared to control women - At 24 months, treatment women were 2.5 percentage points less likely to have ever been pregnant (p <.10). - At 36 months, women aged under 25 years in treatment households had 10 percent fewer births compared to those in control households (p<0.10). | Total 9 parameters;   - Low: 3 - Unclear: 4 - High: 2 |
| 6.1 | Handa et al. 2015  Kenya | Cash transfers for orphans and vulnerable children (CT-OVC) | RCT  Baseline in March to August 2007: 1542 treatment and 755 control households  First follow-up in 2009: 1325 treatment and 583 control households  Second follow up in 2010: 1280 treatment and 531 control households  Third follow up in 2011: Questions on fertility were introduced in this wave; information was gathered from all female aged 12-49. | Ever been pregnant | - Young women in treatment households were 5.5 percentage points less likely to have ever been pregnant as compared to their counterparts in control households (p< 0.05). - The treatment effect remains significant at 4.9 percentage points (p<0.05). | Total 9 parameters;   - Low: 4 - Unclear: 3 - High: 2 |
| 7.1 | Rosenberg et al. 2015  South Africa | Child Support Grant (CSG) | Cohort study; two groups - CSG recipients and non-recipients  Study period: 1998-2008  Sample: 4,845 women (3,392 received CSG, 1,453 did not receive CSG after first birth) | Birth spacing - time to second pregnancy | - Time to second pregnancy was significantly longer among CSG recipients compared to non-recipients at both the 25^th^ [absolute difference (months): 8; 95% CI: 2,14] and 50th percentiles [absolute difference (months): 30; 95% CI: 12, 42] - The association between grant exposure and second pregnancy was 0.66 (95% CI: 0.58, 0.75). Women with first children who aged out of grant eligibility in 2002 had similar second pregnancy rates to women with first children who remained   grant-eligible in 2003 [IRR (95% CI): 0.9 (0.5, 1.4)] | Total 8 parameters;   - Low : 5 - Unclear (Probably yes or probably no): 2 - High: 1 |
